# Medical students’ choices of specialty in The Gambia: the need for career counseling

**DOI:** 10.1186/1472-6920-12-72

**Published:** 2012-08-08

**Authors:** Mustapha Bittaye, Akin-Tunde Ademola Odukogbe, Ousman Nyan, Bintou Jallow, Akinyinka O Omigbodun

**Affiliations:** 1 Department of Obstetrics and Gynaecology, KorleBu University Teaching Hospital, Accra, Ghana; 2 Department of Obstetrics and Gynaecology, College of Medicine, University of Ibadan, Ibadan, Nigeria; 3 Department of Medicine, School of Medicine and Allied Health Sciences, University of The Gambia, Banjul, The Gambia; 4 Department of Medicine, Royal Victoria Teaching Hospital, Banjul, The Gambia

**Keywords:** Medical students, Specialties, Choices, Careers

## Abstract

**Background:**

Understanding preferences for specialties by medical students and the factors driving choices assists policy makers in ensuring optimal spread of personnel across disciplines.

**Methods:**

This cross-sectional survey using self-administered structured questionnaires was conducted on consenting students of the first medical school in The Gambia, established in 1999. Data collection was in June/July 2011. Questions were on sociodemographic characteristics of students, their parents, factors related to career preferences and opinions about counseling services. Data were analysed using JMP 8.0 software.

**Results:**

Respondents were 52.4% of 202 eligible students. Mean age was 24.1 ± 5.0 years. Females constituted 54.7%. Muslims were 72.7% while Gambians formed 77.0%. Commonest specialties chosen by females were Obstetrics/Gynaecology, Paediatrics and Surgery in that order, while males preferred Internal Medicine, Surgery and Obstetrics/Gynaecology. Commonest factors influencing choices by females were ‘focus on urgent care’ (65.5%) and ‘intellectual content of specialty’ (56.9%). For males, these were ‘intellectual content of specialty’ (60.4%) and ‘focus on urgent care’ / ‘individual’s competence’ (50.0% each). More females (30.0%) than males (23.0%) had ever received career counseling, but all students desired it.

**Conclusions:**

Significant gender differences exist in specialty choices and factors influencing these choices amongst these students. All want career counseling.

## Background

Medical education necessitates students studying a wide scope of medical specialties. In recent years, there is a deeper appreciation of career preference in medicine affecting student learning and academic performance. During preclinical and clinical experiences, medical students construct their professional identity through a process of medical socialization [1]. Within this process, they are focused on acquisition of new knowledge and skills, but they also interact with other medical students, health professionals and patients. Through such interactions, students construct their professional identity grounded in principles of the biomedical model [2].

Cultural and societal values also influence future physicians, particularly through student’s interactions with family, friends, and physicians [3]. The process of resolving conflicting views from societal constructs and self-realizations shape students’ views on the types of specialists they want to become. However, not only medical school entrants [4], but even medical school applicants, often have strong preferences for or against some medical careers [5-8].

A lot of researchers have concentrated on the personal characteristics of individuals choosing particular careers [9,10], on background factors in childhood influencing career preferences [11,12], and on associations with particular personality types [13]. Other studies have highlighted the careers of specific groups, such as women doctors [14], attitudes toward specific specialties, such as Psychiatry [15] and Anaesthesia [16,17] and on the basic statistics necessary for workforce planning [18].

Careers differ in their demands, requiring different amounts of intellectual ability, manual skill, long-term commitment, or willingness to work in particular environments [14], and can each be better suited to particular personalities [13], aptitudes, and physical dispositions. Individuals also differ, having different aptitudes, interests and abilities.

Career choice therefore involves people considering the entire range of careers and then circumscribing those which they regard as broadly acceptable, making their eventual choices within that subset [19].

An important practical point highlighted by studies is that choices tend to be negative, meaning that careers are rejected because they do not have attributes which are consonant with the person making the choice, rather than being positively chosen for their special suitability to the person making the choice [19].

Once circumscription has taken place, a number of possible careers still remain. The second stage of choice is compromise. Because of various practical constraints, certain careers are restricted in the number of people they can accommodate or they are unsuitable in other terms, such as their geographical location or the remuneration they can provide. The eventual career chosen is one that 'satisfies' [20], being realistically good, though not optimal. The applications of these theories to medical careers are self-evident and describe many of the problems facing medical students and junior doctors.

Many researchers have demonstrated that career preferences at the time of entering medical school may be a significant predictor of students’ eventual careers and that many students end up in careers that are closely related to their choice of specialty at the beginning of medical school. Thus it is very important to define the factors that affect career specialty in the early years of medical school [7,8]. Career counselling would identify the students’ inclination early and guide them to careers most suitable for them. This will enable medical students find careers that are appropriate for them sooner rather than later [19]. Many doctors devote too much time in their initial postgraduate years toward trying to enter a specialty for which they are either not suited or are insufficiently endowed with the attributes required [21]. This is exemplified by the findings of a study which revealed that, eleven years afterward, only 65% of doctors ended up in the specialty of their first choice as pre-registration house officers [21].

Unavoidable limitations in the breadth of medical school curricula mean that there are some specialties that undergraduates never experience. Only a few medical schools in developing countries have introduced career counseling services for students which give them the opportunity to experiment and explore during elective postings, along the lines of the model offered by the Career Advisory Service of the American Association of Medical Colleges [19]. However, no studies to explore the choice of careers have been undertaken in the medical school in The Gambia.

The objectives of this study were to determine the career preferences, the factors influencing these choices and the need for career counselling in medical students attending the School of Medicine and Allied Health Sciences, University of The Gambia.

## Methods

### Study population

The University of The Gambia (UTG) medical school, located in the capital city – Banjul, was established in 1999 and has graduated three batches of doctors. It has seven (7) classes. The first year (pre-medical) class covers General Sciences. The pre-clinical class introduces the basic biomedical sciences in a modular format. The clinical class covers clinical clerkships, in which the clinical specialties of Surgery, Internal Medicine, Paediatrics and Obstetrics and Gynaecology are introduced along with selected subspecialties such as Radiology, Anesthesia, Dermatology, Ophthalmology, Psychiatry, and Ear, Nose and Throat. During the clinical years three professional examinations are taken and there is an option of having one elective month. The medical school uses Royal Victoria Teaching Hospital for clinical postings.

### Study design

We did a cross–sectional survey with the study population consisting of all the 202 students in the school. One of the investigators (BJ) gave questionnaires to all class presidents for distribution to all class members. After reading and signing the written consent, participants self administered a structured questionnaire to determine their sociodemographic characteristics, choices of specialty, factors influencing these choices and their opinions on the need for establishment of career counseling services. The occupations of their parents were classified according to the U.S.A 2010 Standard Occupation Classification [22]. In The Gambia, customarily the father is responsible for virtually all household expenses while the mother attends to other needs. Thus we considered the father’s occupation having more influence on the school a child attends. Female education is also still generally low, hence the mother’s educational level will impart greatly on the development of the child and the economic status of the family.

### Ethical approval

This was granted by the Joint Research and Publication Committee of the School of Medicine and Allied Health Sciences, University of The Gambia.

### Statistical analysis

Data were analyzed by JMP 8.0 software. The general approach used was to compare the responses of the male to those of the female students. For these analyses, we examined the following basic predictor variables: demographic factors, choice of specialty and factors that influence choice of specialty and opinion on the need for establishment of career counseling services. Results of continuous variables were expressed as mean ± standard deviation. Statistical analysis was done, using the *χ*2-test for all categorized data. Where the chi-square test was not applicable, an appropriate nonparametric test was used. Using nominal logistic regression, we investigated gender differences in the factors likely to contribute or not contribute to career selection among the students. Odds ratios and 95% confidence intervals were calculated. P values less than 0.05 were considered statistically significant.

## Results

### Socio-demographic characteristics of study population

The response rate was 52.4%, 106 students returning the completed questionnaire out of 202. Over half, (53.4%) of the total student population were females. The mean age (±SD) of the respondents was 24.1 (± 5.0) years. Only 27.3% of participants were Christians and the rest were Muslims. The mean age of the fathers of respondents was 58.5 (± 8.7) years and of their mothers was 49.2 (± 9.3) years. The socio-demographic characteristics of the students by gender are described (Table 1). Regarding the response rate and gender, a slight difference between the females (54.7%) as compared to the males (45.3%) was noted, but this was not statistically significant. The female students in the pre-clinical level accounted for more than half of the participants while the male students were predominant among respondents in the clinical classes, with an overall statistically significant difference between the genders at both levels (p = 0.02).

**Table 1:** Socio-demographic characteristics of respondents

	**Total**		**Female**		**Male**		**P-value**
	**N**	**%**	**N**	**%**	**N**	**%**	
Level	106		58	54.7	48	45.3	
First year	13	12.3	9	69.2	4	30.8	0.0200
Second year	30	28.3	21	70.0	9	30.0	
Third year	8	7.6	3	37.5	5	62.5	
Fourth year	24	22.6	12	50.0	12	50.0	
Fifth year	13	12.3	5	38.5	8	61.5	
Sixth year	6	5.7	4	66.7	2	33.3	
Seventh year	12	11.3	4	33.3	8	66.7	
Nationality							
Gambians	77	72.6	44	57.1	33	42.9	0.4136
Others	29	27.4	14	48.3	15	51.7	
High school attended							
Public	49	46.2	20	40.8	29	59.2	0.0077
Private	57	53.8	38	66.7	19	33.3	
Father’s occupation							
Management, professional, related occupation	64	60.4	38	59.4	26	40.6	0.2539
Service occupation	7	6.6	5	71.4	2	28.6	
Sales and office occupation	4	3.8	2	50.0	2	50.0	
Natural resources, construction & maintenance	20	18.9	6	30.0	14	70.0	
Production, transportation & material moving	4	3.8	2	50.0	2	50.0	
Retired	7	6.6	5	71.4	2	28.6	
Mother’s educational level							
Non-formal	42	39.6	16	38.1	26	61.9	0.0217
Less than high school	12	11.3	10	83.3	2	16.7	
High school	19	17.9	12	63.2	7	36.8	
More than high school	33	31.1	20	60.6	13	39.4	
University payment system							
Parents	15	14.1	8	53.3	7	46.7	0.9715
Extended family	16	15.1	8	50.0	8	50.0	
Scholarship	63	59.4	35	55.6	28	44.4	
Others	12	11.3	7	58.3	5	41.7	
Total number of family members							
2 – 4	26	24.5	12	46.2	14	53.9	0.0635
5 – 7	55	51.9	36	65.5	19	34.6	
> 8	25	23.6	10	40.0	15	60.0	
Birth order							
First	30	28.3	16	53.3	14	46.7	0.0741
Second	22	20.8	12	54.6	10	45.5	
Third	22	20.8	16	72.7	6	27.3	
Fourth	12	11.3	8	66.7	4	33.3	
> Fifth	20	18.9	6	30.0	14	70.0	

Statistical analysis was calculated by the *χ*^2^-test.

Gambians accounted for more than two-thirds (77.0%) of the subjects. Nearly 4% of the students had a physician father. Regarding the educational attainment of the mothers of the students, mothers of the females were significantly more educated than the mothers of male students (p = 0.0217). Prior to being admitted into the Medical School, more females (66.7%) attended private schools than the males (33.3%), a statistically significant difference (p = 0.0077).

### Specialty choices

The five most common specialties considered by both sexes were Surgery, Obstetrics and Gynaecology, Internal Medicine, Paediatrics and Public Health. If students were allowed three choices, 66 (62.3%) chose Surgery, 65 (61.3%) chose Obstetrics and Gynaecology and 63 (59.4%) chose Internal Medicine amongst both sexes. The most common specialties considered by female students were Obstetrics and Gynaecology, Pediatrics and Surgery respectively while the males considered Internal Medicine, Surgery and Obstetrics and Gynaecology.

But if only those who chose the subjects as a first choice were examined it was found that 36.2% of females chose Obstetrics and Gynaecology, 21.4% chose Surgery, 20.7% chose Paediatrics and 10.3% chose Internal Medicine while 35.4% of males each chose Surgery and Internal Medicine and 8.3% chose Public Health. Only 6.3% of males chose Obstetrics and Gynaecology. More than 81.0% of males at least considered Internal Medicine compared to 41.4% of females. Table 2 shows the different proportions of the choices made.

**Table 2:** Specialty preferences among medical students by gender and ranking of choices

					**Female (n = 58 54.7 %)**						**Male (n = 48 45.3 %)**					
**Total (n = 106)**	**Total**		**First Choice**		**Second Choice**		**Third Choice**		**Total**		**First Choice**		**Second Choice**		**Third Choice**	
**Variable**	**n**	**%**	**n**	**%**	**n**	**%**	**n**	**%**	**n**	**%**	**n**	**%**	**n**	**%**	**n**	**%**
Surgery	33	56.9	13	21.4	9	15.5	11	19.0	33	68.8	17	35.4	9	18.8	7	14.6
Obstetrics and Gynecology	43	74.1	21	36.2	14	24.4	8	13.8	22	45.8	3	6.3	11	22.9	8	16.7
Pediatrics	34	58.6	12	20.7	16	27.6	6	10.3	21	43.8	2	4.2	11	22.9	8	16.7
Internal medicine	24	41.4	6	10.3	9	15.5	9	15.5	39	81.3	17	35.4	12	25.0	10	20.8
Psychiatry	2	3.4	0	0.0	1	1.7	1	1.7	4	8.3	2	4.2	0	0.0	2	4.2
Orthopedics	3	5.2	0	0.0	0	0.0	3	5.2	4	8.3	0	0.0	0	0.0	4	8.3
Ophthalmology	4	6.9	0	0.0	0	0.0	4	6.9	1	2.1	0	0.0	0	0.0	1	2.1
Dermatology	4	6.9	0	0.0	2	3.5	2	3.5	2	4.2	1	2.1	0	0.0	1	2.1
Anesthesiology	4	6.9	0	0.0	2	3.5	2	3.5	2	4.2	1	2.1	0	0.0	1	2.1
Radiology	3	5.2	2	3.5	0	0.0	1	1.7	3	6.3	1	2.1	1	2.1	1	2.1
Public Health	15	25.9	3	5.2	5	8.6	7	12.1	8	16.7	4	8.3	2	4.2	2	4.2
Family Medicine	4	6.9	0	0.0	2	3.5	3	5.2	3	6.3	1	2.1	1	2.1	1	2.1
Basic sciences	0	0.0	0	0.0	0	0.0	0	0.0	2	1.2	0	0.0	0	0.0	2	4.2
Ear, Nose and Throat	0	0.0	0	0.0	0	0.0	0	0.0	0	0.0	0	0.0	0	0.0	0	0.0

The students showed little interest in the other subjects including Family Medicine. Only one female chose Anesthesia as a first choice and no male at all did so. More than one-third (35.0%) of males chose Internal Medicine as first choice as opposed to 10.3% of females. No female chose Psychiatry as a first choice and only 2 (3.4%) females chose it as second or third choice while 4.2% of males chose it as a first choice. Overall 8.3% of males chose it as one of their choices. No male or female chose ENT in any of their three allowed choices.

Family Medicine was considered by 6.9% of females and chosen by 1 (2.1%) male as a first choice. No male or female chose Orthopedics as a first choice.

### Factors influencing career selection

The most important factor regarded as being strongly contributory to the choice of specialty among female respondents was ‘focus on urgent care’ (65.5%), followed by ‘the intellectual content of the specialty’ (56.9%) and ‘interest in research’ (53.5%). The most important factor that strongly influenced choice of specialty by males was ‘intellectual content of the specialty’ (60.4%) followed by ‘focus on urgent care’ / ‘the individual competencies of the student’ (50.0% each) and ‘lack of specialist in that area’ / ‘interest in research’ (45.8% each).

Table 3 shows the details of the factors affecting the choices of specialty by male and female medical students. There was a significant difference between the genders in some of the factors that they considered significant in influencing their choice of speciality. While 41.7% (95% confidence interval, CI: 31.75 – 51.65) of the males stated that ‘duration of residency programme’ would influence their choice, only 22.4% (95% CI: 13.67 – 31.13) of the females considered this to be a contributory factor (p = 0.0050). Male respondents attached more importance to mentor emulation than females when choosing their careers (p = 0.0383). ‘Lack of specialists in that area’ was regarded as contributing to speciality choice by both males (60.4%) and females (65.5%) but while only 5.2% of the females regarded it as not contributing, 20.9% of the male students stated that it did not influence their choice.

**Table 3:** Factors influencing choice of specialty and their distribution among male and female students

					**Female (n = 58 54.7%)**										**Male (n = 48 45.3%)**						
	**SC**		**MC**		**N**		**MNC**		**SNC**		**SC**		**MC**		**N**		**MNC**		**SNC**		
	**n**	**%**	**n**	**%**	**n**	**%**	**n**	**%**	**n**	**%**	**n**	**%**	**n**	**%**	**n**	**%**	**n**	**%**	**n**	**%**	**P-value**
Lack of specialists in that area?	25	43.1	13	22.4	17	29.3	1	1.7	2	3.5	22	45.8	7	14.6	9	18.8	1	2.1	9	18.8	0.5427
Shorter hours of practice	4	6.9	3	14.6	22	47.9	5	4.2	24	27.1	3	6.3	7	14.6	23	47.9	2	4.2	13	27.1	0.0652
On-call schedule	7	12.1	5	8.6	25	43.1	9	15.5	12	20.7	6	12.5	7	14.6	25	52.1	4	8.3	6	12.5	0.4901
Flexibility of specialty	15	25.9	8	13.8	22	37.9	6	10.3	7	12.1	10	20.8	14	29.2	17	35.4	3	6.3	4	8.3	0.4642
Interaction with other physicians	16	27.6	21	36.2	16	27.6	3	5.2	2	3.5	20	41.7	14	29.2	11	22.9	0	0.0	3	6.3	0.1941
The reputation of specialty	24	41.4	10	17.2	22	37.9	1	1.7	1	1.7	16	33.3	11	22.9	15	31.3	1	2.1	5	10.4	0.3548
The duration of residency program	8	13.8	5	8.6	29	50.0	1	1.7	15	25.9	5	10.4	15	31.3	22	45.8	3	6.3	4	8.3	0.4642
Work pressure	10	17.2	11	19.0	22	37.9	4	6.9	11	19.0	5	10.4	9	18.8	20	41.7	6	12.5	8	16.7	0.7525
Interest in research	31	53.5	9	15.5	12	20.7	4	6.9	2	3.5	22	45.8	14	29.2	10	20.8	0	0.0	2	4.2	0.8909
Interest in long term relations with patients	23	39.7	13	22.4	18	31.0	1	1.7	3	5.2	11	22.9	19	39.6	15	31.3	0	0.0	3	6.3	0.3531
Physician patient interaction	31	53.5	10	17.2	11	19.0	1	1.7	5	8.6	16	33.3	19	39.6	9	18.8	0	0.0	4	8.3	0.2233
Diversity of patients	21	36.2	15	25.9	16	27.6	4	6.9	2	3.5	13	27.1	16	33.3	13	27.1	3	6.3	3	6.3	0.4854
Anticipated income	11	19.0	11	19.0	27	46.6	5	8.6	4	6.9	8	16.7	12	25.0	18	37.5	1	2.1	9	18.8	0.1979
Focus on community health	24	41.4	12	20.7	15	25.9	2	3.5	5	8.6	18	38.3	14	29.8	11	23.4	2	4.2	2	4.2	0.8992
Focus on urgent care	38	65.5	6	10.3	12	20.7	0	0.0	2	3.5	24	50.0	12	25.0	8	16.7	0	0.0	4	8.3	0.1884
The curriculum	19	32.8	15	25.9	22	37.9	0	0.0	2	3.5	12	25.0	14	29.2	17	35.4	1	2.1	4	8.3	0.3433
The intellectual content of specialty	33	56.9	7	12.1	15	25.9	0	0.0	3	5.2	29	60.4	8	16.7	9	18.8	0	0.0	2	4.2	0.5619
That you are likely to stay in the city	12	20.7	3	5.2	26	44.8	4	6.9	13	22.4	5	10.4	7	15.6	22	45.8	2	4.2	12	25.0	0.7238
The individual’s competencies	28	48.3	9	15.5	17	29.3	2	3.5	2	3.5	24	50.0	9	18.8	12	25.0	1	2.1	2	4.2	0.7394
Mentor emulation	14	24.1	10	17.2	26	44.8	3	5.2	5	8.6	17	35.4	14	29.2	13	27.1	2	4.2	2	4.2	0.0383
Advice from faculty members	9	15.5	7	12.1	26	44.8	5	8.6	11	19.0	5	10.4	10	20.8	19	39.6	3	6.3	11	22.9	0.6738
Advice from friends	6	10.3	9	15.5	25	43.1	2	3.5	16	27.6	6	12.5	8	16.7	18	37.5	5	10.4	11	22.9	0.6467
Advice from parents	14	24.1	10	17.2	20	34.5	3	5.2	11	19.0	6	12.5	13	27.1	18	37.5	2	4.2	9	18.8	0.5279
Advice from practicing physicians	15	25.9	12	20.7	23	39.7	1	1.7	7	12.1	12	25.0	10	20.8	17	35.4	2	4.2	7	14.6	0.9368

Statistical analysis was calculated by the *χ*^2^-test**.**SC-strongly contributory, MC-mildly contributory, N-neutral, MNC-mildly non-contributory, SNC-strongly non-contributory.

With nominal logistic regression analysis, ‘lack of specialists in that area’ and ‘mentor emulation’ were the only two factors where the differences between males and females in the issues influencing their choice of careers reached the level of statistical significance (Table 4). The ‘duration of residency programme’ that showed a statistically significant difference in the initial analysis did not show such a difference on logistic regression.

**Table 4:** Univariate logistic regression to isolate gender differences in factors influencing career choice

		**Female vs Male**		
		**Univariate**		
	**OR**	**95 % CI**		**p-value**
‘Lack of specialists in that area’				
Neutral	1.0			
Contributory	0.7	0.3 -	1.8	0.4465
Not Contributory	0.2	0.0 -	0.7	0.0178*
‘Mentor emulation’				
Neutral	1.0			
Contributory	0.4	0.2 -	0.9	0.0292*
Not Contributory	1.0	0.3 -	4.3	1.0000
‘Duration of residency’				
Neutral	1.0			
Contributory	0.5	0.2 -	1.2	0.1201
Not Contributory	2.0	0.7 -	6.4	0.2051

Statistical analysis used: nominal logistic regression.

### Career counselling

More than 90.0% of the subjects said there was no career counselling facility in the university. All respondents thought that there is a need for career counselling unit in the university. About 30.0% of female and 23.0% of male students respectively had received career counselling before in their life. These data are summarized in Table 5.

**Table 5:** Distribution of responses on the need for career counseling among students

	**Female (n = 58 54.7 %)**				**Male (n = 48, 45.3 %)**			
	**Yes**		**No**		**Yes**		**No**	
	**n**	**%**	**n**	**%**	**n**	**%**	**n**	**%**
Do you have career counseling unit in your university?	2	3.5	56	97	3	6.3	45	94
Do you think there is a need for career counseling unit in your Medical school?	58	100.0	0	0.0	48	100.0	0	0.0
Have you ever received career counseling?	17	29.3	41	71	11	22.9	37	77

## Discussion

The University of The Gambia is very young and is yet to produce its own specialists. It will have to make policies on what specialties are priorities and it is thus important to determine the factors affecting the choice of specialty by medical students in The Gambia. This will help avoid the problems of maldistribution of specialists. Surgery, Obstetrics & Gynecology, Internal Medicine, and Pediatrics were the most preferred specialties among medical students of The Gambia medical school in this study.

The most preferred specialty by female students was Obstetrics and Gynecology followed by Pediatrics, Surgery and Internal Medicine. For male students it was Internal Medicine followed by Surgery, Obstetrics and Gynecology and Pediatrics. These findings are similar to those reported in other studies [23-25]. Among female respondents the preferred first choice of specialty was Obstetrics and Gynecology which is similar to a study done in Jordan [25]. This however is very different from that carried out in Kenya where females chose Pediatrics as their first choice (40.0%) followed by Surgery (28.0%) [26]. For the males in our study, both Internal Medicine and Surgery tied for the most preferred first choice of specialty, followed by Public Health and Obstetrics and Gynecology. This is similar to the finding in Kenya where most male students chose Surgery (40.0%) followed by Internal Medicine (15.0%) [26].

The low rate of males choosing Obstetrics and Gynecology is different from what was seen in a study in Nigeria [27] where Obstetrics and Gynecology was their second most chosen subject. This low rate in The Gambia may be due to the country being a predominantly Muslim country.

Many studies have shown males to be more inclined towards Surgery and Internal Medicine [25] but Public Health is not that commonly preferred. The selection of Public Health as third choice by our male students is peculiar. This might be because The Gambia had a School of Public Health many decades before the establishment of the medical school and Public Health officers man several health projects in the Ministry of Health. Medical students may be hoping to take over these projects upon graduation, being better qualified than the present Diploma in Public Health holders.

Unlike in Jordan where Orthopedics was the third most preferred specialty, none of the male respondents in this study chose it as a first choice. This is of major concern as The Gambia needs urgent human resources in the area of Accidents and Traumatology and students need to be encouraged to consider this.

Similar to a study done in Jordan no male chose Anesthesia as a first choice but was considered by 4.2% of females. This can be attributed to the number of nurse anesthetists employed at university hospitals [25]. The teaching hospital in The Gambia has a Diploma programme for nurse anesthetists. Medical students may be reluctant to compete with nurses and thus will explore other fields instead.

The low rate of choice of Basic Medical Sciences is noteworthy as no female considered it and even the two males who did, chose it as their third choice. Previous studies have shown that whenever a specialty is not even considered among the first three choices then it is very unlikely that students would consider it later for specialization [4,8,28]. This is of concern because currently, nearly all the Basic Medical Science lecturers and practitioners at The Gambia Medical School are expatriates. It is necessary to identify the factors that discourage students from this area and rectify them in order to have the required manpower for this field.

Family Medicine was found to be one of the least popular specialties preferred, which is consistent with the findings of other studies [4,29,30]. Medical students in The Gambia must be encouraged to take up Family Medicine so that our health care system can be involved more in health promotion activities, as soon as the minimum required number of specialists to run the medical school has been attained. Many researchers have tried to determine the factors that influence the choice of specialty by medical students [9,10,31]. Both personal and academic (preclinical and clinical) experiences are important in shaping students' perceptions of specialties and their personal choices [9]. Mentoring, a process of giving advice, imparting knowledge or teaching psychomotor skills over a period of time to a junior apprentice by a senior colleague is very important in medical practice, with the former wishing to be like the latter. Some authors have indeed shown ‘mentor emulation or positive interaction’ to be very influential in career choice amongst medical students [9,32].

We have found ‘mentor emulation’ to have been considered significantly differently between males and females, being more influential amongst the former. ‘Mentor emulation’ has been found to be highly rated in Surgery [25] and this is the specialty chosen by majority of the male students. ‘Lack of specialists in that specialty’ was highly rated as being contributory to their choice by both sexes. This is important in The Gambia as the medical school has just started training doctors and there are no indigenous specialists in most of the specialties. Many students are motivated to be the first specialists in their areas of choice. A much higher proportion of the male students than the females, however, did not consider this to be a factor contributing to their choice. This may also be related to the fact that Surgery, the choice of a large number of the male students is already well-represented among the specialists in the institution.

It was surprising to find in our study that a higher proportion of male students found ‘duration of residency program’ a contributory factor to their choice, compared to their female counterparts. This deviates from the expectation that female doctors might wish to finish with residency training more quickly so as to have more time for their families.

The findings of a study in Turkey revealed that the most important reasons for the choice of specialty were “better financial opportunities” and “prestige”, followed by “personal development”, “more benefits for the patient”, and “wish to work in an urban area”. The situation is quite different from the results of our study as both sexes did not rate ‘expected income’ or these other factors high among the factors contributing to their choice.

The most important factors that affected choice of specialty among female respondents were ‘focus on urgent care’ followed by “the intellectual content of the specialty” and then “interest in research”. Because most female respondents chose Obstetrics and Gynaecology as their first specialty, it is reasonable that “focus on urgent care” is rated the most influential factor for their choice of specialty. Female respondents rated “interest in research” as an influential factor more than their male counterparts.

All the students in our study said that there was no career counselling facility in the medical school. Only one quarter (25.0%) of both sexes had received career counselling before. This is not ideal as students are left on their own without guidance including in the choice of their intended specialties. This may lead to a lot of trial and error and may cause delay. Career guidance ought to be offered early, based on evidence and tailored to the characteristics and needs of the individual. Young doctors should be able to recognise the exciting possibilities open to them and base their decisions on positive choice rather than an increasingly demoralising process of elimination [19]. All students agreed that there is need for career counselling in the medical school. But the method of career counselling most appropriate would have to be determined in another study as there are different methods and tools [19].

## Conclusions

This study was undertaken to determine the career preferences of medical students at the University of The Gambia Medical School and the factors influencing these choices. It was also to determine their need for career counselling. The most common specialty chosen by female students was Obstetrics and Gynaecology probably because of the predominant religion in the country while the most influential factor was ‘focus on urgent care’. Males’ predominant choices of specialty consisted of Internal Medicine and Surgery. ‘The intellectual content of the specialty’ was their most influencing factor. The Basic Medical Sciences were not the first or second choice of any student. No male or female chose ENT in any of their three allowed choices.

Less than one third of all students had ever received career counseling and all students regarded career counseling as a necessity. It is recommended that career counseling units be set up in the medical school and students be counseled early in order to assist them choose appropriate careers and also prevent maldistribution of doctors among the specialties. A study to identify the best method of counseling students in this medical school is hereby suggested.

The Government of The Gambia should encourage students to specialize in less preferred areas that are essential in the short term.

This study has limitations as it did not determine the point in their schooling that medical students actually make their decisions in order to know the most appropriate stage to introduce career counselling. The response rate was also not very large. More analysis could have been done with the demographic information but gender was the main outcome measure analyzed.

## Competing interests

We hereby declare that MB submitted a dissertation based on findings from this study in partial fulfillment of the requirement for the award of the Master of Science degree in Biomedical Education of the Faculty of Clinical Sciences, College of Medicine, University of Ibadan, Nigeria in 2011.

## Authors’ contributions

MB conceptualized the study, drafted the proposal, collected and analysed data, and wrote the article. AAO revised the proposal, the analysed data, took part in the write-up and is the correspondence author. ON was involved in the conceptualization, data analysis and write-up. BJ assisted with data collection and analysis. AOO revised the data analysis and assisted in the write-up. All authors read and approved the final manuscript.

## Pre-publication history

The pre-publication history for this paper can be accessed here:

http://www.biomedcentral.com/1472-6920/12/72/prepub
